# Knowledge, Awareness, and Attitudes Regarding Concussion Among Middle Schoolers

**DOI:** 10.7759/cureus.4863

**Published:** 2019-06-09

**Authors:** Thor S Stead, Yasamin Daneshvar, Sarah Ayala, Latha Ganti

**Affiliations:** 1 Emergency Medicine, Alpert Medical School of Brown University, Providence, USA; 2 Podiatry, New York College of Podiatric Medicine, New York, USA; 3 Medical Education, Touro College of Osteopathic Medicine, Vallejo, USA; 4 Emergency Medicine, Envision Physician Services, Orlando, USA

**Keywords:** concussions, adolescents

## Abstract

Introduction: Given the widespread prevalence of concussions in children under the age of 19, the adolescent perspective, as well as an understanding of the mechanisms behind traumatic brain injury (TBI), is extremely important. The authors sought to assess the knowledge, awareness, and attitudes regarding concussion among middle school children and whether a brief educational intervention based on clinical data and science resulted in a change of their knowledge or attitudes towards concussions.

Methods: A 20-question survey design was administered before and after an educational intervention. The surveys were anonymous, but they were paired so that it was possible to correlate the pre- and post-test answers to the respondents. An eighth-grader at a Florida middle school conducted this study after school hours after the student and their parent or legal guardian signed informed consent. This project was approved by the Science Department at Howard Bishop Middle School in Alachua County, Florida. JMP® 14.0 (SAS Institute Inc., Cary, NC) was used for statistical analyses.

Results: The cohort was 64% female, age range: 13 - 15, and consisted of local eighth-grade students. Forty-three percent never wore a helmet when riding a bicycle or skateboard. Only 68% knew that the state had a helmet law for kids. Participants were significantly more likely to feel they had good TBI knowledge after the intervention (p = 0.0005, 95% confidence interval (CI) 0.1937 to 0.6863) and that the didactic lectures changed the way they thought about safety (p = 0.0034, 95% CI 0.1025 to 0.5175). Students reported that their mothers (vs. fathers) were significantly more likely to wear seatbelts (p = 0.05, 95% CI 0.0036 to 0.5036), and they themselves reported wearing seatbelts more often after the survey.

Conclusions: There still exists a knowledge gap when it comes to pediatric concussion. Expanding awareness is important in order to bolster safety measures among adolescents.

## Introduction

Mild traumatic brain injury, also known as concussion, is common in children, with sports and recreation as a leading cause [1-2]. More than 150,000 school-aged children present to the emergency department (ED) each year for concussion and concussion-related injuries in the United States [3]. The number of ED visits and hospitalizations for concussions is also increasing [4]. Given the widespread prevalence of this public health problem, understanding of concussions - why, how, and where they can occur, as well as what should be done about them - is extremely important. The authors sought to assess the knowledge, awareness, and attitudes regarding concussion among middle school children.

## Materials and methods

This was a pre- and post-survey design with a 45-minute didactic educational session in between the pre-test and post-test, designed to assess the effectiveness of such an intervention on the knowledge and attitudes towards concussion or mild traumatic brain injury (TBI), as well as baseline characteristics. The survey instrument collected demographics, information on students’ safety habits, and their knowledge and attitudes as they relate to concussion or mild TBI. The educational intervention involved a Powerpoint® (Microsoft Corp., Redmond, WA) presentation developed by the authors that covered the epidemiology, pathophysiology, and clinical outcomes after a concussion, as well behaviors more likely to result in a concussion. The Powerpoint presentation was followed by a review and distribution of printed materials from the Centers for Disease Control’s Heads Up campaign. The setting was a public middle school in Alachua County, Florida, USA. Students from the eighth grade were recruited to participate as part of the author’s (TSS) science project. A letter explaining the project was sent home with each student, and students could participate after the student and their parent or legal guardian signed the informed consent. The surveys were anonymous, but they were paired so that it was possible to correlate the pre- and post-test answers to the respondents. The surveys were collected in a single sitting at school after regular school hours. The middle school science department approved the project in accordance with local ethics.

Statistical analyses were performed using JMP 14.0 for the Macintosh (SAS Institute Inc., Cary, NC). Descriptive statistics were used to report cohort characteristics, participation in sports, and demographics. For the questions relating to knowledge and attitudes about concussion, percentages were reported for each response in the pre- and post- intervention categories. The two-proportion Z test was used to study differences in pre- and post-test answers.

## Results

**Figure 1:**
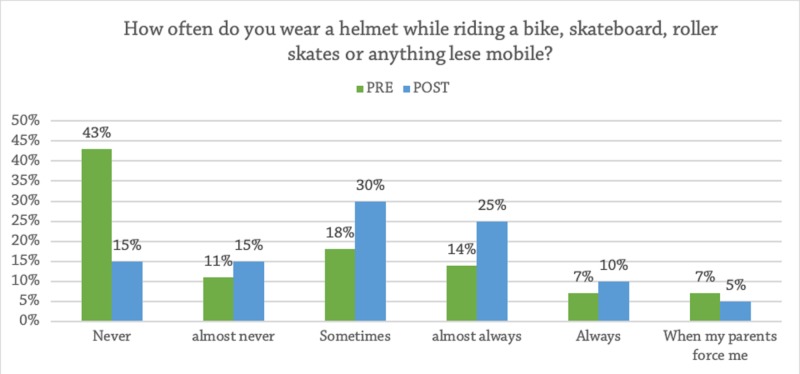
Frequency of Helmet Use

At baseline, 43% of the students never wore a helmet when riding a bicycle or skateboard (Figure 1). Only 68% knew that the state had a helmet law for children. After the educational intervention, 15% said they would still not wear a helmet (p = 0.0228), and 27% learned that Florida does indeed have a helmet law for children (p = 0.0074) (Table 1). 

**Table 1:** Knowledge of Local Helmet Laws CI: confidence interval

Does Florida have a helmet law?				
	PRE	POST	p-value	95% CI
YES	68%	95%	0.0074	0.0726 to 0.4674
NO	32%	5%		

**Figure 2:**
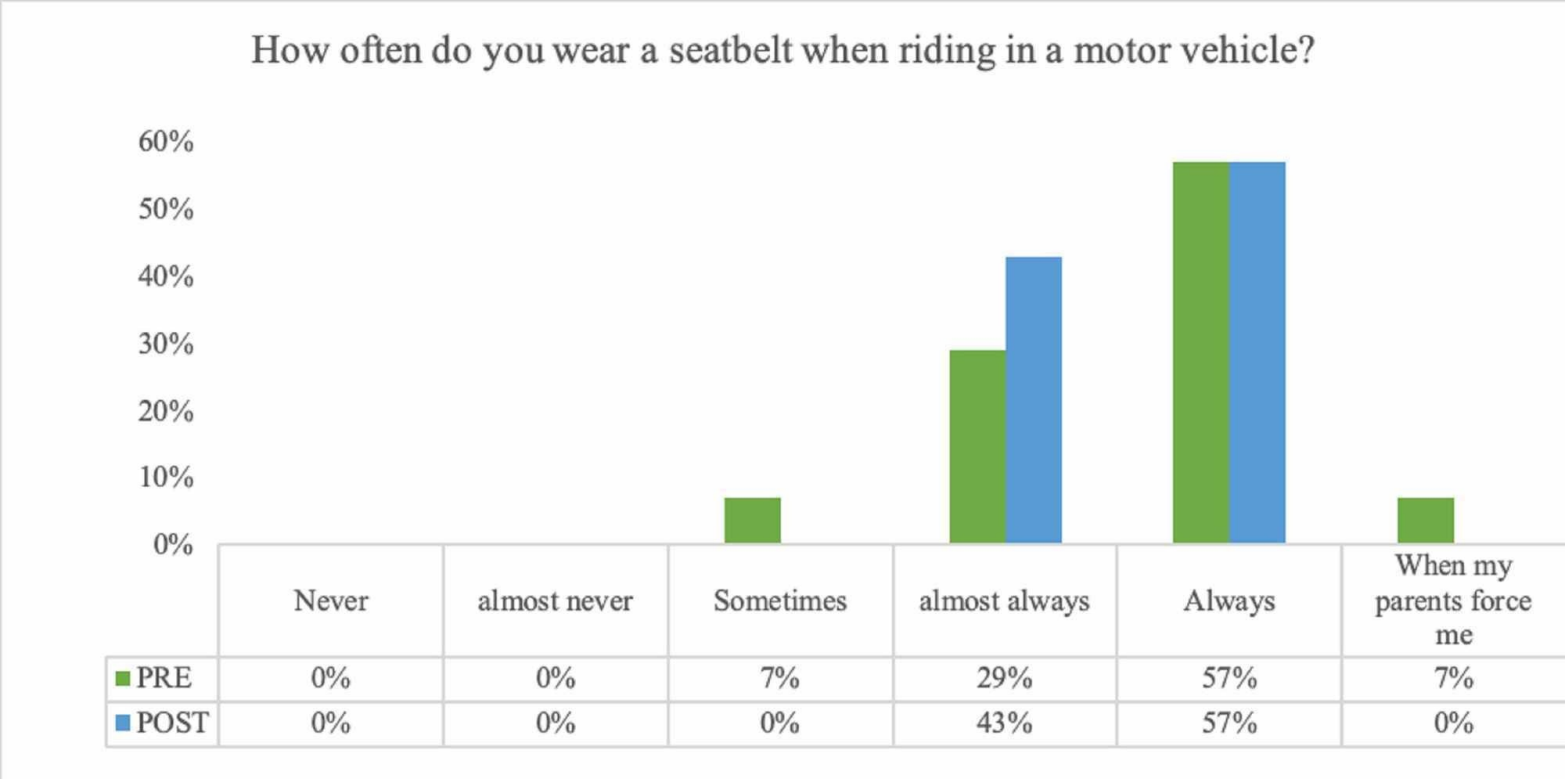
Frequency of Seatbelt Use

**Figure 3:**
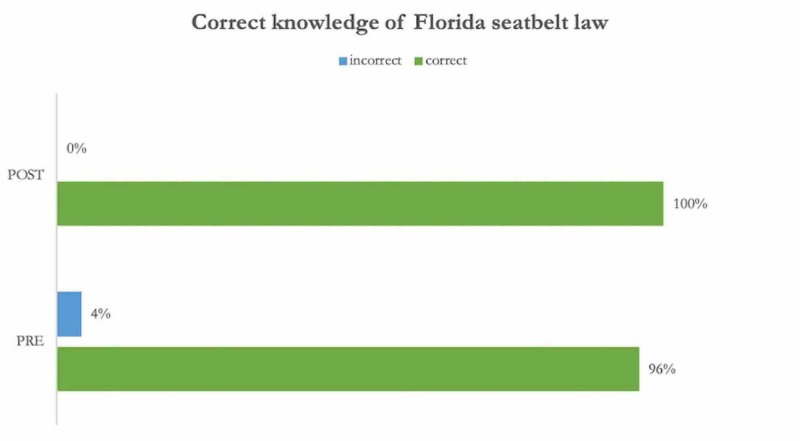
Correct Knowledge of Local Seatbelt Laws for the Driver and Front Seat Passenger

Eighty-six percent of the students reported wearing their seatbelt always or almost always. This number rose to 100% on the post-survey (Figure 2). Students reported that their mothers (vs. fathers) were significantly more likely to wear seatbelts (p = 0.05, 95% CI 0.0036 to 0.5036) (Table 2). Knowledge of seatbelt laws for drivers and passengers is reported in Figure 3.

**Table 2:** Frequency of Seatbelt Use in Parents CI: confidence interval; N/A: not applicable; NS: non-significant

How often does your parent wear a seatbelt?				
	Mother	Father	p-value	95% CI
Never	0%	4%	NS	
Almost never	4%	4%	NS	
Sometimes	7%	14%	NS	
Almost always	14%	18%	NS	
Always	75%	50%	0.05	0.0036 to 0.5036
N/A	0%	11%	NS	

**Figure 4:**
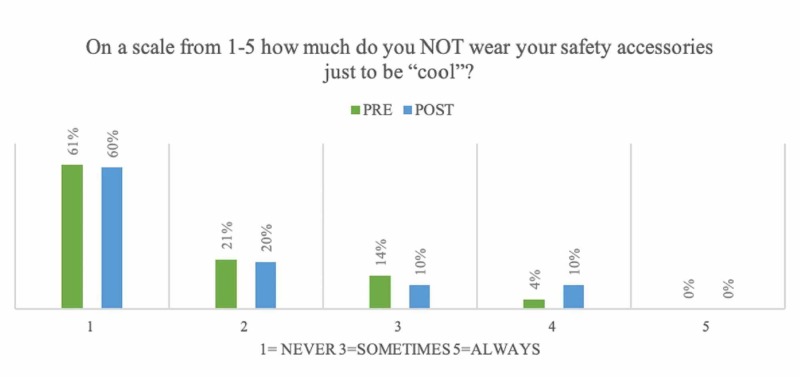
Use of Safety Accessories

**Figure 5:**
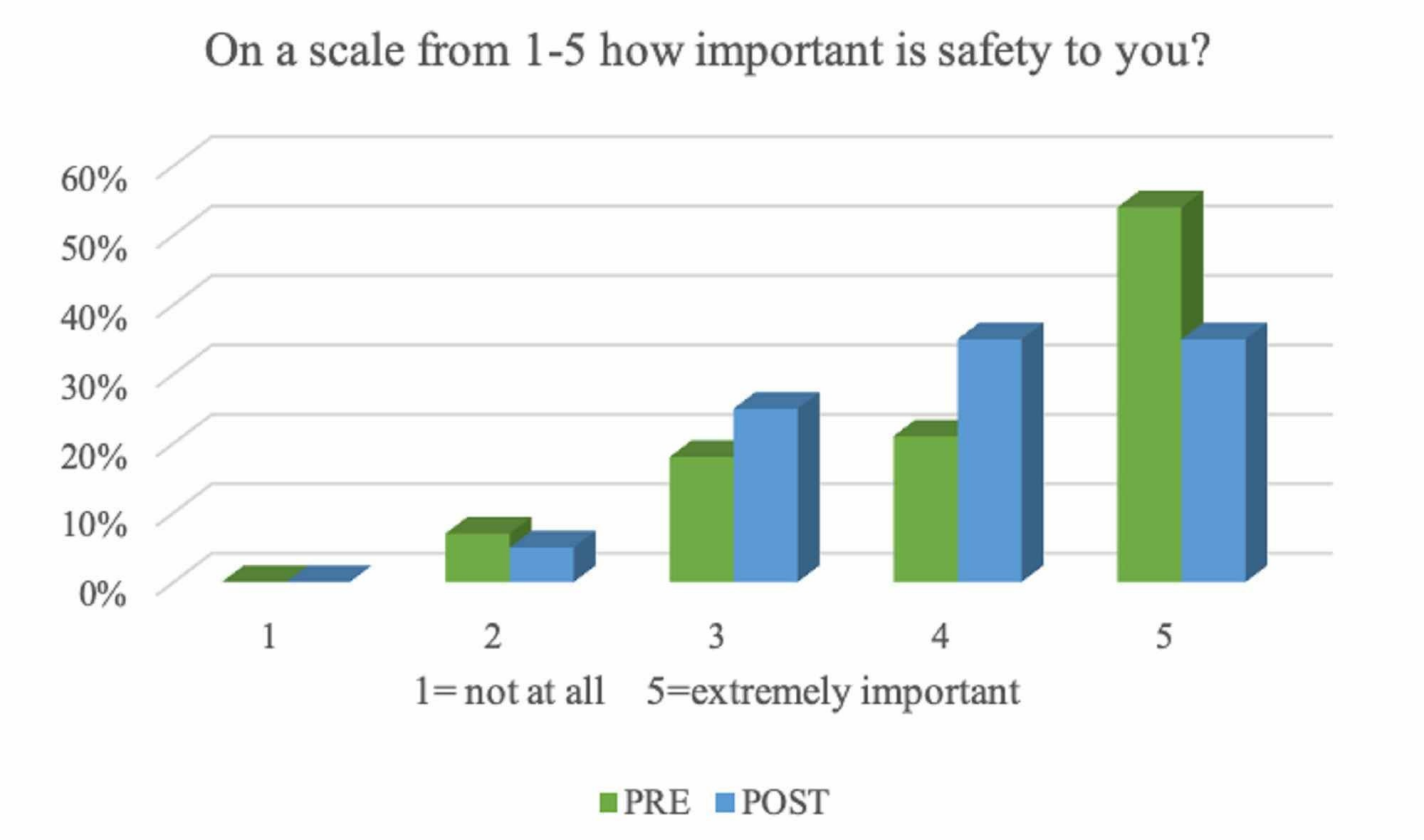
Importance of Safety

Participants’ responses to their use of safety accessories and the importance of safety to them were mixed (Figures 4-5). Participants were significantly more likely to feel they had good TBI knowledge (Table 3) after the intervention (p = 0.0005, 95% CI 0.1937 to 0.6863), and that the didactic lectures changed the way they thought about safety (p = 0.0034, 95% CI 0.1025 to 0.5175). 

**Table 3:** Impact of Safety Lectures CI: confidence interval; N/A: not applicable; NS: non-significant

On a scale from 1 to 5, how much do you think safety lectures and talks change the way you think about safety?				
	PRE	POST	p-value	95% CI
1	29%	5%	0.015	-0.4333 to 0.0467
2	39%	35%	NS	n/a
3	29%	15%	NS	n/a
4	4%	35%	.0060	0.0887 to 0.5313
5	0%	10%	NS	n/a

## Discussion

The literature affirms the knowledge gap demonstrated in this study. One study that examined parents’ knowledge of pediatric concussions in school and on the sports field noted that 51% of respondents were unaware that head trauma was more serious in children, 44% of respondents did not know there were medical guidelines to return to play, and 35% did not know that repeated head trauma could cause dementia [5]. A study of high school athletes (n = 496) found that older child age (p = 0.01) and female sex (p = 0.03) were associated with better knowledge of pediatric concussion [6]. Another article concluded that among student-athletes, athletic trainers were the ones most likely to refer the student to medical help if they suspected a concussion (50.8%). School nurses followed closely (37.0%). Among non-student athletes, guidance counselors were the first ones to respond to suspected concussions (46.5%), followed closely again by school nurses (43.2%) [7].

Another study examined the knowledge gap among coaches and athletic trainers [8] and found that knowledge of symptoms was very good (over 90% of the n = 916 cohort correctly identified five or more of the eight most common symptoms of concussion); however, knowledge of youth susceptibility and repeat concussion was lacking. The study found that less than 40% of the cohort recognized that younger athletes were more susceptible to concussion, and a mere 25% of the subjects knew that subsequent to sustaining a concussion, a repeated head injury can be up to five times as likely to occur. Beyond the knowledge of baseline information about concussions, studies have also examined whether knowledge alone results in behavior change. An anonymous survey of college students (n = 262) (an older cohort than those in the current study) found that 43% of those with a history of concussion reported that they had knowingly hidden symptoms of a concussion to stay in a game and 22% of athletes overall indicated that they would be unlikely or very unlikely to report concussion symptoms to a coach or athletic trainer in the future. These data suggest that there may be a substantial degree of underreporting of concussion among collegiate athletes, despite most acknowledging that they have been formally educated about the risks of concussion [9].

Although many studies have examined this knowledge gap and severity of concussion management in student-athletes and coaches of these athletes, there has been substantially less research on a combined cohort of athletes and non-athletes as part of a junior high cohort, which is what this study examined. We found that pre-presentation knowledge of all aspects of concussion (symptoms, outcomes, mortality, etc.) among students was relatively Gaussian, with no substantial increase or decrease in the knowledge gap between athletes and non-athletes. After the clinical presentation that included mortality and the neurodynamics of a concussion, all students experienced an increase in knowledge and awareness of concussion. Although the mean knowledge level increased almost by a factor of two, a follow-up of students examining safety habits (such as wearing a helmet and seatbelt) was not statistically significant, and no association between knowledge of concussion and changing in safety habits was found. Closing the knowledge gap through clinical and epidemiological presentations of concussion in athletics will hopefully increase the percentage of self-reported and self-diagnosed concussions, thus decreasing the morbidity of concussion and improving outcomes of students all across the nation.

## Conclusions

There still exists a knowledge gap when it comes to pediatric concussion. Expanding awareness is important in order to bolster safety measures among adolescents. Education regarding concussion in the form of school curriculum may help in this regard.
